# “That’s probably how you would want care to be” - experiences of sick leave teams at a health center, a mixed method study

**DOI:** 10.1186/s12875-023-02192-5

**Published:** 2023-11-17

**Authors:** E. Hällås, I. Skoglund, L. Nordeman

**Affiliations:** 1 Research Education Development and Innovation Primary Health Care Region Västra Götaland, Borås, Sweden; 2 Primary Health Care/School of Public Health and Community Medicine, Sahlgrenska Academy, University of Gothenburg, Gothenburg, Sweden; 3 Department of Health and Rehabilitation, Institute of Neuroscience and Physiology, Sahlgrenska Academy, University of Gothenburg, Gothenburg, Sweden

**Keywords:** Sick leave, Sick leave physicians, Rehabilitation coordinator, Teamwork, Primary care

## Abstract

**Background:**

The health center where this study was performed the management wanted to test whether a sick leave team provided the physicians with better conditions for the task of assessing sick leave. The goals were to ensure the quality of the assessment of patients with sick leave needs and to improve the work environment. The aim of this study was to take part in the staff's experiences of having access to and working in sick leave teams and how the working method affected the number of sick leave patients and sick leave pattern.

**Methods:**

A mixture of qualitative and quantitative methods. Two focus groups were conducted with a total of 11 participants. The head of the health center formed the focus groups, which consisted of 6 doctors, 3 district nurses, 1 rehab coordinator and 1 psychologist.

Aggregated sick leave data for full-time and part-time sick leave of more than 90, 180 and 360 days, respectively, were obtained and compiled at project start and end, and from the corresponding period 18 months before project start.

**Results:**

The introduction of sick leave teams with physicians and rehabilitation coordinator for patients who turn to the health center for mental illness and / or musculoskeletal problems emerged three main categories from the analysis of the focus group discussions: working environment, clear roles and in-depth competence.

The total number of people who were on sick leave more than 365 days decreased by 27% between start and the end of the project, and the proportion of women increased by 11%.

**Conclusions:**

The study shows that the complex task of sick leave can be perceived as positive by physicians with the support of teamwork. The working method is similar to that applied in occupational health care, where the physician is not alone with this task. This can also be a way to make primary care a more attractive workplace.

## Background

In Sweden and many other countries, sick leave and its consequences are of great importance to both individuals and society. Sweden has historically had a high level of sick leave and varied over time in a manner that differed from other countries [1].

In health care, a female-dominated sector, factors as job strain, heavy workload and lack of leadership affects the psychosocial work environment and thereby sickness absence and quality of the work performed [2–5]. Therefore, it is essential that the conditions for making assessments of the need for sick leave are good both for patients and employees [6]. Primary care physicians often consider that assessing sick leave is difficult, time consuming, and that complex cases should be handled by specialised colleagues as in the Netherlands, where the employer is responsible for the first two years of rehabilitation and health insurance [6, 7]. Studies show that physicians have difficulties assessing the ability to work with mental illness and predicting return to work [8, 9]. A comparison of five European countries showed that guidelines for support in sick leave assessments co-varied with lower sick leave frequency [10]. In Sweden, medical insurance decision support was introduced in 2007 with guidance for physicians to assess work ability and recommendations for sick leave for various medical diagnoses [11]. Since 2019 Sweden has held legislation on coordinating efforts in case of sick leave. Coordination consists of personal support, internal coordination, and collaboration with other parties [12].

From a European perspective, Sweden has a primary care with a lower proportion of general practitioners (GPs). On the other hand, Sweden has invested in larger health centers with a wider range of health and medical care, more professions and more teamwork, which sometimes causes problems with continuity and collaboration [13].

The physician's tasks of examining, diagnosing and treating are often perceived as difficult to reconcile with the role of medical expert for assessing work ability [14]. At the health center where this study was performed the management wanted to test whether a sick leave team provided the physicians with better conditions for the task of assessing sick leave. The goal was to see the effect on the work environment and sick leave pattern on group level when introducing a sick leave team. The study was done to deepen the knowledge of the working method.

### Aim

The first aim of the study was to investigate obstacles, opportunities, and effects on the work environment since the sick leave team was introduced. The second aim was to describe the change in the number of people on sick leave, the extent and duration of diagnoses in mental illness and long-term pain.

### Participants and settings

To achieve the purpose of sharing knowledge and experiences and changes in sick leave a mixed methods design was chosen [15]. The method was used to gain a broader knowledge of how qualitative and quantitative factors were affected by the new way of working.

The sick leave team at the health center consisted of two specialists in general medicine and a rehabilitation coordinator (RC). Rehabilitation coordination is a new function in health care for providing personal support for people on sick leave and contributes to internal and external coordination of rehabilitation efforts to promote return to work [16]. For the implementation of the project the GPs´ service factors were increased by 0.8 and doubled for the RC to 0.8. The team worked together for two days weekly, and the physicians´ visits were extended to one hour. Patients who expressed a need for sick leave and applied due to mental illness or long-term pain were triaged by a nurse to an RC for a telephone assessment, no later than the following day. If necessary, the RC could book physician visits or arrange visits to another professional group. The occupational therapist mapped activity ability and conditions for return to work.

The working days began with a patient review and ended with an assessment of the need for continued coordination efforts. The rehabilitation coordinator then provided the patients with support, including contacting employers and authorities if necessary, for rehabilitation and return to work.

### Sample

In the qualitative part focus group interviews were used. After oral and written information the head of the health center formed two groups and written consent was obtained. Of a total of 24 employees, 11 informants participated, average age 45 years (31–63) and average active professional years 17 (5–41), two men and nine women; four specialists in general medicine, two of whom also worked as sick leave physicians, two resident physicians, three district nurses, a psychologist, and an RC. Half of the physicians and a third of the nurses participated in the interviews. The rehabilitation coordinator participated in both focus groups. Deskriptive data of the participants in the focus groups are presented in Table 1.

**Table 1:** Deskriptive data of the participants in the focus groups

Focus group	Gender	Profession	Number of years in the profession
1 and 2	Woman	Physiotherapist (PT) /Rehabilitation coordinator (RC)	PT 27/RC 4
1	Woman	General practitioner	10
1	Man	Resident physician in family medicine	15
1	Man	Resident physician in family medicine	5
1	Woman	District nurse	13
1	Woman	District nurse	24
2	Woman	District nurse	18
2	Woman	General practitioner	12
2	Woman	General practitioner	41
2	Woman	General practitioner	13
2	Woman	Psychologist	13

### Data collection

#### Qualitative data

The focus groups lasted 1.5 h and were conducted at the workplace two weeks in a row. After the introduction, the group leader presented open-ended questions on which the conversations would be based:

- Can you tell us about the experience of working with sick leave teams?
- Can you tell us how your roles have changed by the new way of working?
- Can you discuss how it has affected the work environment?

The conversations, which were lively and perceived as open, were recorded. A deputy group leader kept field notes.

#### Quantitative data

Aggregated sick leave data for full- and part-time sick leave more than 90, 180 and 360 days, respectively, were obtained from Inera Certificate Statistics, a national statistics service for the follow-up of sick leave patterns. Data was compiled at the project’s start (2017–09-01) and completion (2019–02-28) and from the corresponding period 18 months before project start (2016- 03–01 through 2017–08-31).

### Data processing

#### Qualitative data

The recorded interviews were processed using qualitative content analysis with an inductive approach according to Graneheim and Lundman [17]. Interviews were transcribed verbatim by the group leader and listened to several times and summarized to gain an overall picture of the content, both the more obvious and the more hidden, latent message. Meaning-bearing units were identified, merged, coded, and condensed jointly by the group leader and the deputy group leader. Data was compiled into main categories with underlying subcategories and an overall theme [18].

#### Quantitative data

Sick leave data was reported as the number and proportion of full- and part-time sick leave more than 90, 180 and 360 days, respectively. Data was presented for the total group as well as for women and men. The number of medical certificates for sick leave was reported per physician and sick leave physician at project start and conclusion. Windows Excel was used for analyses and graphs.

## Results

### Qualitative results

Three main categories emerged in the analysis of the focus group discussions, working environment, clear roles, and in-depth competence. Fearless leadership was emphasised as an important factor in enabling the work. The courage to make decisions about changing the work situation without a large basis for decision-making created a workplace with structure and routines, well-being, and good staffing. The working method has also provided an increase in skills that has contributed to improved professionalism, security in professional roles and collegial support (Table 2).

**Table 2:** Description of themes, categories and subcategories

**Theme**	**Satisfaction and pride in the teamwork**		
**Categories**	**Working environment**	**Clear roles**	**In-depth competence**
**Subcategories**	Bold leadership with a clear structure and organization	Responsibility and mandate provide security in their professional roles	In-depth learning and development individually and in groups
	Job satisfaction, well-being and predictability	Trust and respect for each other's competence	Increased professionalism within the team and in patient meetings
	Increased collaboration and improved communication	Better resource utilization	Make visible the need for supervision and competence development
	Reduced stress and frustration	Ambivalence to change	

### Working environment

The common perception in the focus groups was that access to the sick leave team had improved cooperation and communication at the health center. This was perceived as positive in relation to job satisfaction, well-being and predictability for the employees. Bold leadership was highlighted leading to a decision on the establishment of the sick leave team despite cost increases for the investment. The stable staffing and competence in the group made the project possible and resulted in a distinct sick leave process that created assurance and predictability for employees and patients:

“I think that a health center based on firm relationships has gained a lot. The patients and the staff feel secure: everyone knows each other.” (Inf 11)

Previously, assessment of sick leave was booked at emergency physician appointments despite awareness of the lack of time, continuity for patients, and a poor working environment. Time and resources were now provided for the sick leave physician. The physicians were also given extended time for unlisted patients with a long medical history.

The team emphasised the importance of concentrating the work to specific days for a specific patient group, creating a team spirit and focus on the task. The establishment of the sick leave team also resulted in reducing the patient group from other physicians, which freed time for new areas of responsibility and developmental work:

“For those of us who had not previously been part of the sick leave team, it has provided relief in our daily routines eliminating interruptions of our half-hour sessions amidst everything else.” (Inf 3)

The approach was described as inclusive, and also perceived as contributive to better resource utilization, even if these effects were not measured:

*“I personally think that greater collaboration makes me feel less lonely than when we first started with this work. It’s a boost to my work environment, absolutely.”* (Inf 1)

The rumour about the working method spread and improved collaboration, and simplified communication with the Swedish Social Insurance Agency (SSIA), the social services, and employers in the area:

*“I don’t see …. the *difficulties* with the SSIA. We’re fortunate to work steadily with three of the same administrators.”* (Inf 2)

The nurses stated clearly that the new way of working, of informing about the sick leave team and referring the target group to the RC, made the conversations easier and less stressful. The number of calls to the health center also decreased when the sick leave team patients received a direct number to the RC:

*"Now I think there’s a big difference when you can just hand over the matter to our RC. I think it makes *telephone* counseling a lot easier. Because previously the matter would remain unresolved until some physician felt they could take over.”* (Inf 7)

The change also affected the RC's work environment and clarified assignments. The manager's support and the extra time enabled development, initiated internal cooperation, and the target group with coordination needs was reached.

In the conversations concerns arose that the present number of sick leave physicians would need to be increased. This increase was to ensure continuity, competence, and stability in staffing, and what could be considered a reasonable number of days per week to cope with the task. To establish a stable structure, they started with two physicians, with thoughts of later spreading the method and role. Guidance and support were requested to cope with the suggested change.

### Clear roles and in-depth skills

The professionalism and competence of the sick leave team was perceived as a major asset that relieved other staff members and was beneficial to the patients. The team itself expressed how the task of sick leave had become interesting and stimulating:

*"… That's probably how you would want care to be."* (Inf 2)

Teamwork improved synchronisation of the rehabilitation efforts and consensus in communication with the patient. It also increased a feeling of belonging, respect, and knowledge of each other´s competence and an understanding of the complexity of the sick leave process:

*“We have grown considerably in our roles through that way of working. Previously, we had little contact. We didn’t know each other even though we had worked together for 4–5 years. Now, as a result, it’s become more open.”* (Inf 5)

Increased insurance medical knowledge, clear routines and access to an RC and the sick leave team instilled the nurses with greater security in their professional roles, and clarity in communication with patients:

*"Yes, you know what to tell them: that you have an idea, a plan for them as soon as they register. They are no longer given an acute appointment, put on two weeks sick leave and then just return for an acute appointment with a new physician and so on."* (Inf 9)

The approach clarified the role of the RC in the sick leave process and was perceived as being in charge. The RC also coordinated planning and information about the sick leave team's patients and contacts with SSIA and employers and also held the role of contact person for the patients on sick leave. Here, personal qualities and skills, and a clear, structured approach that the group considered a requirement for the assignment, were emphasised.

By focusing on the assignment of physician on the sick leave team the group's perception was that the ability to assess, determine length, and prognosticate sick leave had improved along with the ability to delimit the assignment. The collaboration with the RC and access to other competencies at the health center enabled the sick leave physicians to capture the complexity of each sick leave case:

*“I also experience a greater certainty now. Already the first time you meet a patient, you know that… no, this one needs longer full-time sick leave, and for this one I can construct a plan."* (Inf 2)

For the physicians who were not part of the sick leave team, the new way of working meant a substantial change. They no longer met patients who were expected to be on sick leave for longer periods and saw the team's competence as an asset to the health center and considered the working method effective:

*"It´s a huge advantage to have such a team: having competent, experienced people, who could act as consultants."* (Inf 8)

One aspect highlighted was that those not part of the sick leave team lost competence in medical insurance issues and experience of complex sick leave. Several emphasised that the reason for choosing primary care as a specialty was the variation in patients from the cradle to the grave:

*"In a way, it felt a bit like you might be standing on the sidelines and watching because of limited sick leave decision experience, thus losing competence.*” (Inf 6)

The group expressed a need for competence development, as the time had so far been spent on developing the work model, and now replenishment and time for reflection were needed. The need for guidance also emerged since many sick leave cases were linked to mental illness and a difficult life situation. The physician's role in sick leave work was perceived as vulnerable, despite the availability of the sick leave team:

*"It is above all the patients in the worst condition. One is terrified that something serious will happen."* (Inf 4)

### Quantitative results

**Fig. 1:**
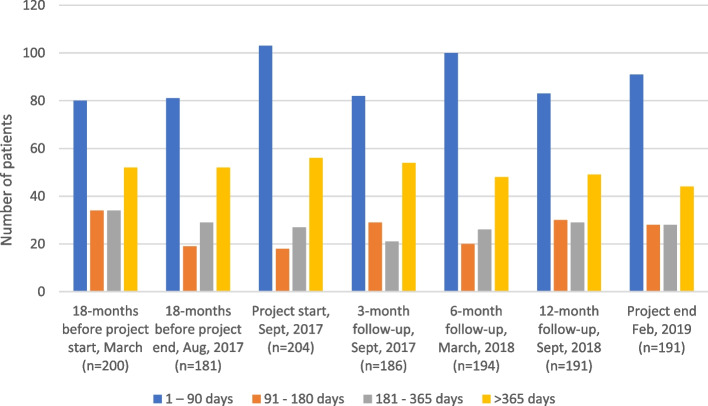
Sick leave length, before, during and after intervention

Two hundred and four people were on sick leave of varying lengths and degree at the start of the project. After 18 months, this figure was 13 less (*n* = 191) (Table 3). The number of people on sick leave within the interval 91—180 days increased by 10 (36%; 10/28) between project start and end (Table 3), while the number of people on sick leave > 365 days decreased by 12 (27%; 12/44) (Table 3, Fig. 1). The proportion of women on sick leave > 365 days increased from 66% at the start of the project to 77% at the end (Table 4).

**Table 3:** Sick listing before, during and after intervention

	18 months before project start March, 2016 (*n* = 200)	18 months before project end Aug, 2017 (*n* = 181)	**Project start** **Sept, 2017** **(*n* = 204)**	3-month follow-up Dec, 2017 (*n* = 186)	6-month follow-up March, 2018 (*n* = 194)	12-month follow-up Sept, 2018 (n = 191)	**Project end** **Feb, 2019** **(*n* = 191)**
*Sick leave duration*							
1 – 90 days	80 (40%)	81 (45%)	**103 (51%)**	82 (44%)	100 (52%)	83 (43%)	**91 (48%)**
91—180 days	34 (17%)	19 (10%)	**18 (9%)**	29 (16%)	20 (10%)	30 (16%)	**28 (15%)**
181—365 days	34 (17%)	29 (16%)	**27 (13%)**	21 (11%)	26 (13%)	29 (15%)	**28 (15%)**
> 365 days	52 (26%)	52 (29%)	**56 (27%)**	54 (29%)	48 (25%)	49 (26%)	**44 (23%)**
*Sick leave grade*							
25%	17 (9%)	14 (8%)	**12 (6%)**	17 (9%)	12 (6%)	17 (9%)	**23 (12%)**
50%	44(22%)	25 (14%)	**32 (16%)**	33 (18%)	31 (16%)	33 (17%)	**27 (14%)**
75%	13 (6%)	9 (5%)	**6 (3%)**	6 (3%)	4 (2%)	12 (6%)	**13 (7%)**
100%	126 (63%)	133 (73%)	**154 (75%)**	130 (70%)	147 (76%)	129 (68%)	**128 (67%)**

**Table 4:** Sick listing in women before, during and after intervention

	18-months before project start March, 2016	18-months before project end Aug, 2017	**Project start** **Sept, 2017**	3-month follow-up Dec, 2017	6-month follow-up March, 2018	12-month follow-up Sept, 2018	**Project end** **Feb, 2019**
Total number of people on sick leave (women % (n/n))	125/200 (63%)	119/181 (66%)	**136/204 (67%)**	127/186 (68%)	136/194 (70%)	129/191 (68%)	**130/191 (68)**
Sick leave duration (women % (n/n))							
1 – 90 days	49/80 (61%)	50/81 (62%)	**70/103 (68%)**	55/82 (67%)	68/100 (68%)	56/83 (67%)	**60/91 (66%)**
91—180 days	21/34 (62%)	14/19 (74%)	**11/18 (61%)**	22/29 (76%)	16/20 (80%)	20/30 (67%)	**18/28 (64%)**
181—365 days	23/34 (68%)	21/29 (72%)	**18/27 (67%)**	15/21 (74%)	18/26 (69%)	16/29 (55%)	**18/28 (64%)**
> 365 days	32/52 (62%)	34/52 (65%)	**37/56 (66%)**	35/54 (65%)	34/48 (71%)	37/49 (76%)	**34/44 (77%)**
*Sick leave grade* women % (n/n)							
25%	14/17 (82%)	9/14 (64%)	**8/12 (67%)**	12/17 (71%)	6/12 (50%)	13/17 (76%)	**18/23 (78%)**
50%	33/44(75%)	14/25 (56%)	**16/32 (50%)**	20/33 (61%)	21/31 (68%)	22/33 (67%)	**15/27 (56%)**
75%	9/13 (69%)	6/9 (67%)	**5/6 (83%)**	6/6 (100%)	4/4 (100%)	10/12 (83%)	**9/13 (69%)**
100%	69/126 (55%)	90/133 (68%)	**107/154 (69%)**	89/130 (72%)	105/147 (71%)	84/129 (65%)	**88/128 (69%)**

**Fig. 2:**
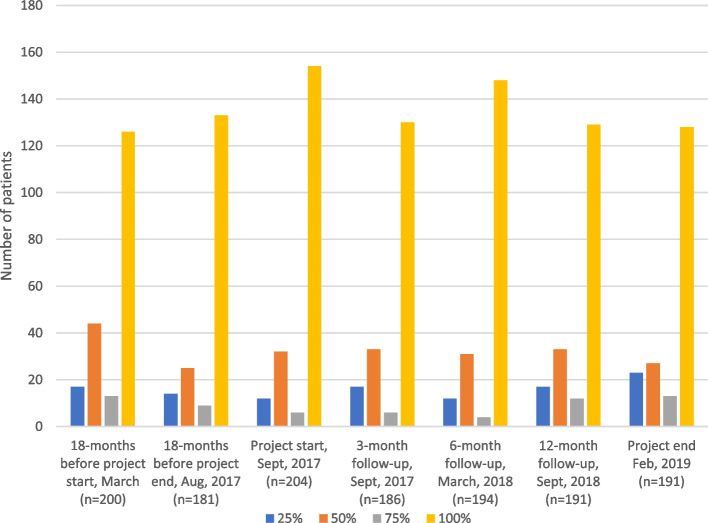
Sick leave grade before, during and after intervention

The number of people on 25% sick leave increased by 11 (49%; 11/23), and the number of people on 75% sick leave increased by 7 (54%; 7/13) between project start and end (Table 3, Fig. 2). The number of people on 100% sick leave decreased by 26 (20%; 26/128) between project start and end (Table 3, Fig. 2). The same pattern was seen for women. However, the proportion of women on full time sick leave at project end was unchanged (Table 4).

The distribution of the number of sick leave cases to sick leave physicians and other physicians changed between project start and end. At the start of the project, the sick leave physicians together had 55 cases (24%) of the total number of cases (*n* = 231), and at the end 100 cases (47%) (*n* = 212). The change in the number of physician visits for sick leave physicians and other physicians between project start and end could not be reported.

## Discussion

The quality analysis of this study shows that sick leave teams with sick leave physicians, extended RC function together with one hour of physician visits contains three main categories; a perceived good working environment, clear roles, and in-depth competence.

The working method has led to in-depth knowledge and confidence in the competence of different professional categories, which has resulted in an experience of better resource utilisation and quality assurance of the internal work with sick-listed patients. Quantitative data showed minor changes in the number of people on sick leave, duration, and extent.

### Results discussion

#### Working environment

The transition to a team-based approach for patients who seek care at the health center due to mental illness and long-term pain and who express a need for sick leave has had a positive impact on the work environment. Working in inter-professional teams has been desired by many professional categories in healthcare but complicated to organise by placing demands on leadership and staffing [19, 20]. Studies of teamwork in primary care usually describe physicians and nurses and their difficulties with roles and collaboration. There are few studies on how effective multidisciplinary teams work, but time consumption of the working method has been criticised: that the division of responsibilities can be unclear, and the difficulty of engaging physicians affects the team's legitimacy [21]. Today, care is complex, which necessitates effective internal and external collaboration, and is also in demand by patients [20]. This health center has succeeded with the teamwork through clear leadership and structure, where knowledge is shared and deepened among team members, which has made the workplace attractive. In order to build safe and efficient teams time is needed to establish the working method, trust and good communication, as this and other studies show [20, 22]. Thus, the question arises whether training should be introduced earlier in medical education.

Because the sick leave physicians have been able to concentrate on the assessment of the need for sick leave with support from the RC and access to rehabilitation teams, they now view their task positively. Occupational health physicians in Sweden have better organisational conditions for this task compared with GPs [23]. This study shows that primary care can be organised so that the assessment of sick leave can also be performed with quality and satisfaction.

The nurses' experience was that the number of, and time for telephone calls, decreased when working with sick leave teams, as patients also received the direct number to the RC. The responsibility for arranging a physician's appointment to retain sickness benefits is a stressful task. Swedish studies show that telephone counselling, which often accounts for 50% of nurses' working hours, is experienced as stressful, unsatisfactory and a work environment problem. The nurse's lack of experience, limited knowledge of insurance medicine, and the high number of calls increased the difficulties [24]. Structural conditions such as routines, internal cooperation and training in insurance medicine emerge as solutions to the problem.

The newly established position of rehabilitation coordinator, often a nurse with several roles, can create ambiguity and stress [12]. However, at this health center the RC had a background in rehabilitation. In this context, coordination is highlighted as an important support for physicians and nurses, with a positive impact on the work environment, which is confirmed in a report made by the Stockholm County Council [16].

#### Roles and skills

The role as a sick leave physician has provided in-depth competence in the form of knowledge and experience, which has increased the skill of investigating and treating complex sick leave cases linked to mental illness and long-term pain. Comparisons can be made with a study showing occupational health physicians having better conditions for the task compared with GPs by receiving time and organisational support from rehabilitation teams [23, 25]. This team-based approach also provided an in-depth knowledge of factors that affect people's conditions and incentives to work. Research shows that assessing work ability and the need for sick leave is complex and that physicians here rely more on their non-medical skills than in ordinary clinical work [14]. Herein also lies the difficulty of dealing with dual roles, of being both a therapist and assessing work ability [14]. This study shows that longer physician visits, teamwork and well-prepared patients gave the physician better conditions to assess sick leave needs and forecast return to work. Teamwork involves, to some extent, a redistribution of work tasks, and the use of the professionals' accumulated knowledge can give the patient added value in their encounter with healthcare [26].

Many GPs wish they could be relieved of long, complicated sick leave cases and invoke the Netherlands' system of far-reaching employer liability and occupational health care [7]. This study shows that several of the physicians wanted the patient variation that exists in primary care. There was a concern about losing competence in insurance medicine among those who were not a part of the sick leave team. However, the working method showed it was possible to combine the breadth of primary care with an in-depth knowledge, and that this was compatible with the GP’s role through teamwork and collaboration.

The study also showed that the nurses had deepened their insurance medical skills and developed a clear approach to patients about sick leave routines at the health centre, which has made conversations with patients simpler. The nurse is usually the first care contact in sick leave matters, and it is important that the meeting leads to the right action for patients who express a need for sick leave. This requires prerequisites such as time, insurance medical knowledge and internal collaboration [27].

The RC was described as a central figure that held together contacts and information about patients on sick leave. Through extended time for coordination and as part of the sick leave team the role and function of the RC was clarified at the health centre. This role included collaborating with different actors within as well as outside health care, for example employers, which has been unusual in Swedish health care. This places demands on leadership so that its function receives support, legitimacy and has the skills needed for the assignment. Since the role of the RC is under development in Swedish healthcare there are ongoing studies evaluating its effects on return to work [28, 29]. A Norwegian cohort study showed no covariation with coordination and previous return to work [30]. In international studies, the role is often linked to rehabilitation programs for return to work, and the client is usually an employer, insurance company or another authority, while in Sweden it is a statutory assignment for health care [31]. The differences make it difficult to draw conclusions about the effects of coordination. However, this study shows that coordination can take place in a structured way based on a described insurance medical process in primary healthcare and in accordance with the legislation [12].

#### Sick leave statistics

The health centre did not systematically follow the changes in sick leave statistics, but their feeling was that the working method was effective. The results showed that the total number of people on sick leave at the health centre decreased by 13, and 12 fewer patients were on sick leave more than a year after the end of the project compared with the start. The proportion of women on sick leave for more than a year increased by 11%. The results point to the difficulty of assessing the risk of long-term sick leave and the impact on return to work even with a structured team effort and sick leave physician. Long-term sick leave is complex often with both medical causes and environmental factors affecting the opportunities to return to work [32]. Both statistics and research show that the majority of those on sick leave are women, and in the present study the proportion of women over time was 2/3 of the total number of people on sick leave. The differences were not affected by the changed way of working. This reflects sick leave in both Sweden and Europe and the difficulties in achieving an equal assessment of women and men in order to reduce unjustified gender differences [33]. Women's ill-health in connection with long-term sick leave is to a large extent related to psychiatric diagnoses [25]. In order to achieve a sustainable return to work, collaboration is needed between actors in the sick leave process and committed employers [32].

Reducing the number of full-time sick leave was one of the goals of the developmental work, and full-time sick leave decreased, but the proportion of women was unchanged. Research points to both the advantages and disadvantages of part-time sick leave and how it affects the return to work, and shows that collaboration with employers and timing are especially important in mental illness [32, 34].

### Method discussion

#### Strengths

Mixed methods provided a broader knowledge and deeper understanding of employees' experiences of the working method, and at the same time a description of changes in sick leave rates. The researchers in the study had extensive experience of work in primary care and sick leave issues based on different professions, and thus a pre-understanding of the context. Focus groups are time-consuming, but good tools in qualitative research. The categories that crystallised recurred throughout the analytical process, performed by the same researchers who conducted the interviews, which can be considered a strength [35]. To give credibility to the material, the researchers held discussions and reflected throughout the analysis.

Two focus groups with a total of 11 informants of 24 employees could be perceived as scant material, but the group with the necessary experience requested was limited. Thus the selection should be considered adequate. This, in combination with how the groups were composed, was judged to provide good insight into how this type of organisational structure and working methods were perceived. The focus groups were conducted two weeks in a row to reduce the risk of influence between groups, the conversations were lively and inclusive, with positive and negative views.

#### Weaknesses

Content analysis with an inductive approach is a time-consuming method with considerable information to process. It is important to credibility for the reader to understand how the essence emerged [18]. The risk that interesting information from the conversations was not coded and categorised cannot be ruled out.

Changes in sick leave data can be difficult to follow and evaluate because of a stepwise shift in sick leave rates. The data contains more medical certificates issued per month than there are patients on sick leave, which may be due to several physicians issuing medical certificates to the same patient during the same month. These incidents are not visible in the certificate statistics.

## Conclusions

The study shows that the complex task of sick leave can be something that the physician finds stimulating. By forming a sick leave team, introducing one-hour physician visits and a structured way of working with specific days of the week teamwork with high competence and an experience of offering patients better care was created. The working method is similar to that described in occupational health care, where the physician is not alone with the task of sick leave decisions but cooperates with licensed employees in assessment and treatment. The change was perceived to have a positive effect on the work environment for all occupational categories involved and the experience was that the patients were satisfied. This can be a way to make primary care an attractive workplace and facilitate the recruitment of employees. How patients experienced meeting a sick leave team needs further research.

The study shows that long-term sick leave decreased between project start and finish. If this is a pattern that can be seen over a period longer than 1.5 years further study is needed.

## Data Availability

The datasets used and/or analysed during the current study available from the corresponding author on reasonable request.
